# Afadin Downregulation by *Helicobacter pylori* Induces Epithelial to Mesenchymal Transition in Gastric Cells

**DOI:** 10.3389/fmicb.2018.02712

**Published:** 2018-11-09

**Authors:** Miguel Sardinha Marques, Joana Melo, Bruno Cavadas, Nuno Mendes, Luísa Pereira, Fátima Carneiro, Ceu Figueiredo, Marina Leite

**Affiliations:** ^1^i3S – Instituto de Investigação e Inovação em Saúde, Universidade do Porto, Porto, Portugal; ^2^Ipatimup – Institute of Molecular Pathology and Immunology, University of Porto, Porto, Portugal; ^3^Department of Pathology, Faculty of Medicine, University of Porto, Porto, Portugal; ^4^Instituto de Ciências Biomédicas Abel Salazar, University of Porto, Porto, Portugal; ^5^Department of Pathology, Centro Hospitalar São João, Porto, Portugal

**Keywords:** *Helicobacter pylori*, Afadin, epithelial to mesenchymal transition, cell–cell junction disruption, gastric cancer

## Abstract

Afadin is a cytoplasmic protein of the adherens junctions, which regulates the formation and stabilization of both the adherens and the tight junctions. Aberrant expression of Afadin has been shown in cancer and its loss has been associated with epithelial-to-mesenchymal transition (EMT). EMT is characterized by the change from an epithelial to a mesenchymal phenotype, with modifications on the expression of adhesion molecules and acquisition of a migratory and invasive cell behavior. While it is known that *Helicobacter pylori* disrupts the tight and the adherens junctions and induces EMT, the effect of the bacteria on Afadin is still unknown. The aim of this study was to disclose the effect of *H. pylori* on Afadin and its impact in the induction of an EMT phenotype in gastric cells. Using two different cell lines, we observed that *H. pylori* infection decreased Afadin protein levels, independently of CagA, T4SS, and VacA virulence factors. *H. pylori* infection of cell lines recapitulated several EMT features, displacing and downregulating multiple proteins from cell–cell junctions, and increasing the expression of ZEB1, Vimentin, Slug, N-cadherin, and Snail. Silencing of Afadin by RNAi promoted delocalization of junctional proteins from the cell–cell contacts, increased paracellular permeability, and decreased transepithelial electrical resistance, all compatible with impaired junctional integrity. Afadin silencing also led to increased expression of the EMT marker Snail, and to the formation of actin stress fibers, together with increased cell motility and invasion. Finally, and in line with our *in vitro* data, the gastric mucosa of individuals infected with *H. pylori* showed decrease/loss of Afadin membrane staining at cell–cell contacts significantly more frequently than uninfected individuals. In conclusion, Afadin is downregulated by *H. pylori* infection *in vitro* and *in vivo*, and its downregulation leads to the emergence of EMT and to the acquisition of an aggressive phenotype in gastric cells, which can contribute to gastric carcinogenesis.

## Introduction

*Helicobacter pylori* is the most prevalent chronic infection worldwide, with almost half of the human population being infected by this bacterium (Zamani et al., 2018). All individuals infected with *H. pylori* develop chronic inflammation of the gastric mucosa, which in some cases may progress through a cascade of alterations that culminate in gastric cancer (Polk and Peek, 2010). In fact, *H. pylori* is regarded as the major risk factor for gastric cancer development, and has been considered as a class I carcinogen by the World Health Organization (IARC, 1994, 2011).

Gastric mucosal inflammation and the development of more severe clinical outcomes of *H. pylori* infection have been attributed to variation of virulence factors between different strains. Among them, the type 4 secretion system (T4SS)-translocated CagA oncoprotein and the VacA cytotoxin are the best recognized, and infection with strains harboring the most pathogenic variants of these factors are associated with greater intensities of gastric inflammation, and with increased risk for developing gastric premalignant lesions, and gastric cancer (Atherton et al., 1995; Figueiredo et al., 2002; Gonzalez et al., 2011).

In the stomach, *H. pylori* can be found in the mucus and in close contact with the epithelium, with a tropism for cell–cell junctions (Tan et al., 2009; Bugaytsova et al., 2017). This proximity of *H. pylori* to intercellular contacts, leads to disruption of the epithelial apical junctional complex (AJC), which includes the tight junctions (TJs) and the adherens junctions (AJs) (Amieva et al., 2003; Wroblewski et al., 2009, 2015; Hoy et al., 2010).

The TJs contribute to the regulation of epithelial paracellular permeability and to maintenance of cell polarity, and are constituted by transmembrane proteins, such as occludin, claudins, and junctional adhesion molecules (JAMs), and by cytoplasmic-associated proteins, like *zonula occludens* 1 (ZO-1) (Zihni et al., 2016). The AJs are located below the TJs, function mainly in cell–cell adhesion, and are composed by the E-cadherin-catenins and by the nectin-Afadin complexes (Takai et al., 2008a; Zihni et al., 2016).

Afadin (AFDN, AF6 or MLLT4) is an actin-binding protein that associates with nectins at AJs, and transiently with ZO-1, and that regulate the formation and stabilization of the junctional complexes (Ikeda et al., 1999; Zhadanov et al., 1999; Yokoyama et al., 2001; Fukuhara et al., 2002; Lorger and Moelling, 2006; Takai et al., 2008b). A growing body of evidence suggests that Afadin is involved in carcinogenesis. In addition to reports of loss of Afadin expression in epithelial-derived breast, colon, and pancreas tumors (Letessier et al., 2007; Sun et al., 2014; Xu et al., 2015), its downregulation led to increased cell invasion *in vitro* and to accelerated tumor growth in mice (Fournier et al., 2011). Furthermore, Afadin was shown to be a negative regulator of the epithelial-to-mesenchymal transition (EMT) marker Snail in pancreatic cancer (Xu et al., 2015).

Epithelial-to-mesenchymal transition describes the differentiation of epithelial cells into mesenchymal cells, and is an important process during embryogenesis, organ development, tissue regeneration, and cancer progression (Kalluri and Weinberg, 2009). EMT is characterized by loss of the AJC, where junctional proteins are degraded or delocalized, the cortical actin cytoskeleton is reorganized with the formation of lamellipodia and filopodia, and there is repression of cytokeratin intermediate filaments and expression of vimentin filaments (Lamouille et al., 2014). Accompanying these morphological changes, there is reprogramming of gene expression through activation of the mesenchymal phenotype regulators, such as Snail, Slug, and zinc-finger E-box-binding homeobox 1 (ZEB1) transcription factors, concomitantly with downregulation of epithelial markers (Lamouille et al., 2014).

Along with the changes in expression and localization of proteins of the AJC, *H. pylori* infection is able to increase cell invasive properties (Oliveira et al., 2006; Costa et al., 2016), and to activate several signaling pathways that induce an EMT phenotype in the infected cells (Saito et al., 2010; Yin et al., 2010; Baud et al., 2013; Bessede et al., 2014; Lee et al., 2014; Yu et al., 2014; Wroblewski et al., 2015). Therefore, we aimed to determine the effect of *H. pylori* infection on Afadin and its impact in the induction of an EMT phenotype in gastric cells.

## Materials and Methods

### Cell Culture and Transfections

The human gastric cancer cell lines MKN74 (a kind gift from Carla Oliveira, University of Porto) and NCI-N87 (ATCC^®^ CRL-5822^TM^), were cultured in RPMI 1640 (Biowest), supplemented with 10% fetal bovine serum (HyClone^TM^, GE Healthcare Life Sciences) and with 100 U-100 μg/mL penicillin-streptomycin sulfate (Gibco^®^), at 37°C, under a 5% CO_2_ humidified atmosphere.

Cell transfections were performed using the Lipofectamine^®^ 2000 transfection reagent (Invitrogen^TM^ Life Technologies), according to the manufacturer’s protocol. An siRNA silencing Afadin expression (sc-43007, Santa Cruz) and a negative control siRNA (All Stars Negative Control siRNA; QIAGEN, Germany), were used at a final concentration of 75 nM in serum- and antibiotic-free Opti-MEM medium (Invitrogen). The efficiency of transfection was evaluated by Western blot.

### Bacterial Strains and Growth Conditions

*H. pylori* strain 26695 (ATCC 700392, *cag*PAI+, *vacA* s1/m1) was obtained from ATCC (LGC Standards, United Kingdom), and *H. pylori* strain 60190 (ATCC 49503 *cag*PAI+, *vacA* s1/m1) and its respective 60190CagA^-^, 60190CagE^-^, and 60190VacA^-^ mutants were a kind gift from Professor John Atherton (University of Nottingham). Strains were cultured in Trypticase^TM^ Soy Agar with 5% Sheep Blood (TSAII; Becton, Dickinson and Company) at 37°C under microaerophilic atmosphere (GENbox microaer; bioMérieux S.A.) for 48 h, as previously described (Costa et al., 2016). For the production of conditioned media, bacteria were grown in F12 medium (Gibco) supplemented with 1x cholesterol (Gibco) under microaerophilic conditions at 37°C with constant rotation (150 rpm) over 24 h. Subsequently, bacterial suspensions were centrifuged at 15,000 × *g*, for 15 min, and the supernatants were filtered through a 0.2-μm sterile filter, and concentrated by ultrafiltration using a 10 kDa pore size Amicon (EMD Millipore). The resulting concentrated bacterial conditioned media was used as media for gastric cell cultures in comparison with F12 medium plus 1x cholesterol alone without bacteria, as control.

### Infection of Gastric Cells

Gastric cell lines were grown in antibiotic-free medium at 100% confluence for 5 days in 6-well plates (TPP^®^ Plastic Products AG). Medium changes were carried out every day. For infection experiments, bacteria grown for 48 h were collected in phosphate buffer saline (PBS, pH 7.4) and added to gastric cell monolayers, at a multiplicity of infection (MOI) of 100 bacteria per cell. Co-cultures were maintained for 16 h at 37°C, under a 5% CO_2_ humidified atmosphere. Uninfected control cell cultures were processed similarly, with the addition of PBS instead of bacteria. For studies with bacterial conditioned media, F12 medium supplemented with 1x cholesterol (Gibco) alone or after 24 h of bacterial growth was used as culture medium.

### Western Blot and Antibodies

Cells were collected and lysed in 1% Triton X-100, 1% NP-40 in PBS, pH 7.4, containing a cocktail of inhibitors of proteases (Roche Applied Science, Mannheim, Germany) and of phosphatases (Sigma-Aldrich). After supernatant recovery, the protein concentration was assessed using the Bio-Rad protein assay kit (Bio-Rad), according to the manufacturer’s protocol. Identical amounts of protein were subjected to SDS-PAGE and transferred onto nitrocellulose membranes (Amersham). Membranes were blocked with 5% fat-free milk for 1 h, at room temperature, incubated overnight with primary antibodies at 4°C, and incubated with secondary antibodies for 1 h, at room temperature. Antibodies were as follows: anti-AF6 (610732) and anti-β-Catenin (610153) were from BD Transduction Laboratories^TM^; anti-Vimentin (5741), anti-N-Cadherin (13116), anti-Snail (3879), anti-Slug (9585), anti-TCF8/ZEB1 (3396), and anti-E-Cadherin (3195) were from Cell Signaling Technology; anti-GAPDH (sc-47724) was from Santa Cruz Biotechnology; anti-E-cad HECD1 (13-1700), anti-ZO-1 (61-7300), and anti-Occludin (71-1500) were from Thermo Fisher Scientific; anti-αTubulin (T9026) was from Sigma-Aldrich; and horseradish peroxidase-conjugated secondary anti-rabbit (NA934) and anti-mouse (NA931) were from GE Healthcare Life Sciences.

### Transwell Matrigel Invasion Assay

Matrigel-coated 24-well invasion inserts with 8 μm pores (Corning^TM^, BD Biosciences) were used for the *in vitro* invasion assay. Upon hydration with RPMI medium, cells were seeded at a density of 5 × 10^4^ cells in RPMI with 10% FBS, on top of the Matrigel in the upper side of the insert; the bottom side was filled with culture medium. For infection experiments, *H. pylori* was added at a MOI of 100. After 24 h incubation, non-invading cells were removed with a cotton swab, the remaining cells were fixed in methanol for 10 min on ice, and the filter was mounted with Vectashield^®^ with DAPI (Vector Laboratories). The whole filter was counted, using a 20× magnification.

### Immunofluorescence

Cells were grown on glass coverslips (Marienfeld, Germany), fixed with 4% paraformaldehyde or ice-cold methanol. Upon permeabilization and blocking with 5% goat serum, 0.3% Triton X-100 in PBS, for 1 h, at room temperature, primary antibody was added for 2 h at room temperature. Coverslips were then washed several times with PBS pH 7.4, before incubation with the respective Alexa Fluor-conjugated secondary antibodies (Invitrogen). Coverslips were mounted with Vectashield^®^ with DAPI, and fluorescence was monitored in a Zeiss Axio Imager Z1 Apotome microscope.

### Transepithelial Electrical Resistance (TER) and Fluorescein Isothiocyanate (FITC)-Dextran Permeability Assay

Cells were seeded at 100% confluence in 6.5 mm Transwell^®^ with 3.0 μm Pore Polyester Membrane Insert (Corning). Integrity of the epithelial monolayer was determined using a Millicell ERS Voltohmmeter (Millipore). Procedures of electrode equilibration and decontamination were performed according to the manufacturer’s instructions.

Transepithelial electrical resistance (TER) was measured every 24 h, for six consecutive days. For infection experiments, *H. pylori* was added at MOI of 100, and TER was measured every 24 h, for two consecutive days. To avoid temperature influence, plates were allowed to reach room temperature for 15 min, and medium was changed after each measurement. TER values were calculated as ohms/cm^2^. On the sixth day post Afadin silencing, and the second day post infection paracellular permeability was assessed by measuring the permeability of the cell monolayer to FITC-dextran (FD4, Sigma). After measuring the TER, the medium was changed in the inner and bottom chamber, and allowed to equilibrate for at least 1 h. One mg/mL of FITC-dextran was added to the inner chamber and incubated at 37°C for 1 h. After incubation, fluorescence was measured at an excitation of 485 nm and emission of 544 nm, on a Synergy Mx microplate reader. The amount of diffused dextran was determined using calibration curves established with the stock solution. Medium without FITC-dextran was used as blank.

### Single Cell Motility

For evaluation of single cell motility, 12-well plates (TPP^®^ Plastic Products AG) were coated with 5 μg/mL of Fibronectin (Biochrom), overnight at 4°C. Plates were washed with sterile PBS pH 7.4, and 1.5 × 10^3^ cells were added to each well, allowing them to adhere for 16 h. Adherent cells were labeled with 5 μg/mL of Vybrant^®^ CM-Dil Cell-Labeling Solution (Thermo Fisher Scientific) over 20 min, followed by three washes with warmed RPMI without phenol red media. Ten different areas were selected per well, and live images were captured every 15 min on a Leica DMI 6000 time-lapse microscope (Leica Microsystems, Wetzlar, Germany). The migratory paths and velocity for each cell were analyzed with the Manual Tracking plugin function available at the Fiji software package^1^.

### *AFDN* Gene Expression in Normal *H. pylori-*Infected and Non-infected Stomachs

RNA-seq reads sequenced in stomach samples from 181 individuals without disease were obtained from the Genotype-Tissue Expression (GTEx) database (Consortium, 2015). Non-human unmapped reads were aligned against a dataset of 197 bacterial whole genomes collected from NCBI, comprising species identified in the gastrointestinal tract by the Human Microbiome Project and additional species appearing in disease conditions (Turnbaugh et al., 2007). Bacterial quantification and normalization were estimated using the QmihR pipeline (Cavadas et al., 2017). A sample was considered as infected by *H. pylori* when counts of this species reached a log_2_ cutoff value of 5. mRNA expression data for the *AFDN* gene, in reads per kilobase per million (RPKM), was extracted from GTEx release v6.

### Patient Materials and Histopathology

Forty-two formalin-fixed and paraffin-embedded gastric tissue specimens were retrospectively retrieved from the Department of Pathology of Centro Hospitalar S. João (CHSJ; Supplementary Table 1), from patients undergoing bariatric surgery that had specimens obtained for histopathologic examination (mean age [±SD], 40.4 ± 12.6 years; female to male ratio, 6:1). Samples have been used retrospectively, and have been delinked and unidentified from their donors. The study was approved by the ethics committee of CHSJ.

Specimens were stained with hematoxylin/eosin and modified Giemsa, the latter for *H. pylori* detection. Histological assessment evaluated the following parameters: *H. pylori* infection, chronic inflammation, polymorphonuclear activity, glandular atrophy, and intestinal metaplasia. Histological slides were examined by an experienced pathologist, blinded for the clinical information of the patients.

### Immunohistochemistry

Immunohistochemistry was performed in a LabVision Autostainer 480S (Thermo Scientific). Briefly, slides were deparaffinized in xylene and hydrated through passages in 100%, 95%, 70% ethanol, and rinsed in water. Antigen unmasking was performed for 35 min using 1x antigen retrieval solution from the Enzo Polyview^®^ Plus HRP-DAB (anti-Rabbit) kit. Slides were cooled for 20 min and washed with TBS-Tween-20, 0.01%. Endogenous peroxidase activity was blocked for 10 min, and slides were washed and placed in the Autostainer. After blocking of endogenous peroxidase activity, slides were incubated with Antibody blocker/diluent (provided in the kit) for 10 min to prevent unspecific binding, followed by 1 h incubation with primary polyclonal antibody Anti-AFDN (HPA030213, Sigma), 1:1250 diluted in antibody diluent (Thermo Scientific, Labvision). To prevent non-specific binding, slides were again incubated with antibody blocker/diluent for 5 min. After wash, the Polyview Plus HRP (anti-Rabbit) was added and incubated for 1 h. The reaction was revealed with 3,3′-diaminobenzidine (DAB) 5 min. Counterstain was performed with HIGHDEF^®^ hematoxylin (ENZO).

### RNA Extraction and Quantification of Afadin Expression by Quantitative Real-Time PCR

Total RNA was extracted using *mir*Vana^TM^ miRNA Isolation Kit (Thermo Fisher Scientific) following manufacturer’s instructions from uninfected and 24 h-infected MKN74 cells. RNA was reversed-transcribed using M-MuLV Reverse Transcriptase and random-hexamers (NZYTech). Quantification of Afadin expression was performed by quantitative real-time PCR (RT-qPCR) using the TaqMan Gene Expression Assays (Applied Biosystems), *Hs00984486_m1* for Afadin and *Hs99999905_m1* for GAPDH, as endogenous control gene, on an ABI Prism 7000 Sequence Detection System (Applied Biosystems), in four independent experiments. Data were analyzed by the comparative 2^(-ΔΔCT)^ method (Livak and Schmittgen, 2001) and Wilcoxon signed-rank test applied.

### Statistical Analysis

The unpaired Student’s *t*-test was used for comparisons between two independent groups, and the one-way ANOVA with *post hoc* Tukey’s test was used for comparisons between three independent groups. Kruskal–Wallis test was applied to GTEx data that compared AFDN mRNA expression between infected and non-infected samples. Wilcoxon signed-rank test was applied to quantitative real-time PCR analysis. The relationship between the presence of *H. pylori* and alterations to the immunohistochemical pattern of Afadin was assessed using the Fisher’s exact test.

Data analysis was performed using GraphPad Prism Version 6.0. Statistically significance was set at *P* ≤ 0.05 (^∗∗∗∗^*p* ≤ 0.0001, ^∗∗∗^*p* ≤ 0.001, ^∗∗^*p* ≤ 0.01, ^∗^*p* ≤ 0.05). Data in graphs represent mean ± standard error of the mean (SEM) of at least three experiments.

## Results

### *H. pylori* Infection Decreases Afadin Expression

Alterations to several TJ and AJ proteins induced by *H. pylori* infection have been reported (Amieva et al., 2003; Hoy et al., 2010), but the influence of *H. pylori* on Afadin is so far unknown.

**FIGURE 1:**
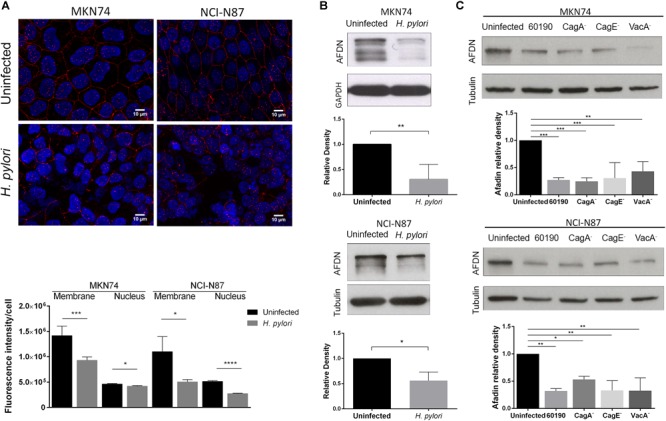
Impact of *H. pylori* infection on Afadin expression. MKN74 and NCI-N87 gastric cell lines were left untreated or infected with *H. pylori* 26695 **(A,B)** or infected with *H. pylori* 60190, or with the respective CagA^-^, CagE^-^ and VacA^-^ mutants **(C)**, for 24 h at a MOI of 100. Afadin detection by immunofluorescence (red) and quantification of the fluorescence intensity at the membrane and the nucleus **(A)**, and by Western blot and its quantification, using GAPDH or tubulin as loading controls **(B,C)**. Scale bar, 10 μm. Data correspond to the mean value ± SEM and are representative of at least three independent experiments. Statistical significance was evaluated with the Student’s *t-*test or with one-way ANOVA.

To investigate the effect of *H. pylori* on Afadin, MKN74 and NCI-N87 cell lines that establish competent junctional complexes, were infected with strain 26695 for 24 h. Immunofluorescence analysis showed Afadin present both at the membrane and in the nucleus in uninfected conditions. Upon infection with *H. pylori* there was a significant decrease in Afadin staining in both cell lines, and at both membrane and nucleus subcellular localizations, as determined by imaging quantification (Figure 1A). Within the nucleus, a significant decrease in the number of Afadin nuclear dots was also observed in infected cells compared to uninfected gastric cells (Supplementary Figure 1A).

Afadin has two major isoforms, the l-Afadin isoform that contains an F-actin-binding domain, and the less expressed s-Afadin isoform that lacks the F-actin-binding domain (Buchert et al., 2007). Western blot analysis with an antibody that recognizes the two Afadin isoforms revealed the presence in both cell lines of a highly expressed 220 kDa protein, corresponding to l-Afadin, and faint bands above the 150 kDa marker, corresponding to s-Afadin. Silencing of Afadin by siRNA confirmed the loss of the two isoforms (Supplementary Figure 1B). Upon infection with *H. pylori* 26695, there was a significant decrease of the protein levels of Afadin (Figure 1B), without changes in the mRNA expression levels (Supplementary Figure 1C).

To evaluate the impact of the major bacteria virulence factors on *H. pylori*-mediated Afadin downregulation, cells were infected with *H. pylori* strain 60190 and its respective CagA^-^, CagE^-^ (required for a functional T4SS), and VacA^-^ mutants. As previously observed in infections with strain 26695, *H. pylori* 60190 was also able to significantly reduce Afadin protein levels in the two cell lines (Figure 1C). Infections with all of the *H. pylori* mutant strains also led to significant decreases in Afadin expression meaning that loss of Afadin is independent of CagA, VacA, and any factor delivered *via* the T4SS (Figure 1C). To determine if secreted soluble bacterial factors or viable bacteria are involved in loss of Afadin, either conditioned medium of *H. pylori* liquid cultures or heat-killed *H. pylori* were incubated with epithelial cells. The results revealed that loss of Afadin protein expression was dependent on the presence of viable bacteria but that was not triggered by soluble bacterial factors secreted by *H. pylori* (Supplementary Figure 1D).

### *H. pylori* Induces an EMT Phenotype in Gastric Cells

Epithelial-to-mesenchymal transition is characterized by the transition from an epithelial to a mesenchymal phenotype, with the modification of the adhesion molecules expressed by the cell, allowing it to adopt a migratory and invasive behavior (Nieto et al., 2016). It has been shown that *H. pylori* is able to modify several cell–cell adhesion molecules (Fedwick et al., 2005; Fiorentino et al., 2013; Schmidt et al., 2016; Tegtmeyer et al., 2017), to induce an elongation in the cell shape (Moese et al., 2004; Bourzac et al., 2007), to increase cell invasion (Oliveira et al., 2006; Costa et al., 2016), and to up-regulate EMT gene expression (Yin et al., 2010; Baud et al., 2013). *H. pylori* was also shown to alter Snail localization in both MKN28 cells, which form functional TJs, and in human gastric specimens (Wroblewski et al., 2015). Still, the great majority of these results were obtained using gastric cancer cell lines that have deficient AJs due to mutations in the E-cadherin-encoding gene, as is the case of AGS cells. Therefore, we wanted to know if *H. pylori* could recapitulate some of these properties in our gastric cell line models that establish proficient AJs.

**FIGURE 2:**
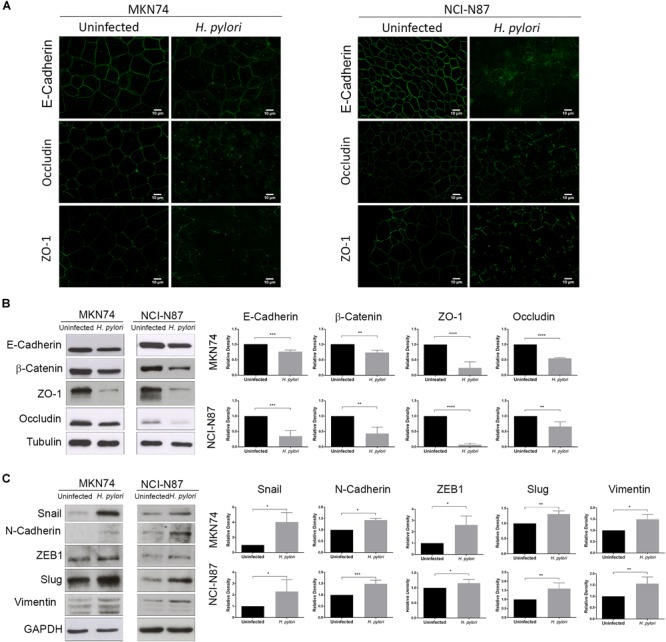
*H. pylori* induces an EMT phenotype in epithelial gastric cells. MKN74 and NCI-N87 gastric cell lines were left untreated or infected with *H. pylori* 26695 for a period of 24 h at a MOI of 100. **(A)** Immunofluorescence of E-cadherin, occludin, and ZO-1. Scale bar, 10 μm. **(B)** Western blot and respective quantifications of the levels of apical junctional complex proteins E-cadherin, β-catenin, ZO-1, and occludin. Tubulin was used as loading control. **(C)** Western blot and respective quantifications of the levels of mesenchymal markers Snail, N-cadherin, ZEB1, Slug, and Vimentin. GAPDH was used as loading control. Data correspond to the mean value ± SEM and are representative of three independent experiments. Statistical significance was evaluated with the Student’s *t-*test.

Evaluation of the effects of *H. pylori* on cell monolayers, showed that after 24 h of infection, there was a decrease in the expression and/or membrane displacement of E-cadherin, occludin, and ZO-1 (Figure 2A). Concurrently, *H. pylori* infection significantly reduced E-cadherin, β-catenin, occludin, and ZO-1 protein levels, both in MKN74 and NCI-N87 cell lines (Figure 2B). Furthermore, *H. pylori* infection significantly increased the protein levels of ZEB1, Vimentin, Slug, N-cadherin, and Snail in both cell lines (Figure 2C).

These results confirm that in addition to Afadin, *H. pylori* infection is able to alter junctional complexes, dampening epithelial cell properties. These results further show that *H. pylori*-mediated increase in the expression of EMT-associated markers also occurs in cell lines that establish AJs.

### Afadin Downregulation Delocalizes AJ and TJ Proteins, Promotes the Formation of Actin Stress Fibers, and Upregulates the EMT Marker Snail

To mimic the effect of *H. pylori* infection on Afadin downregulation and to evaluate the effect of the loss of Afadin in gastric cells, the next experiments were performed with cells that were transiently transfected with a siRNA for Afadin or with a non-silencing siRNA control. Efficient knockdown was achieved in MKN74 (87%) and in NCI-N87 (54%) cells.

**FIGURE 3:**
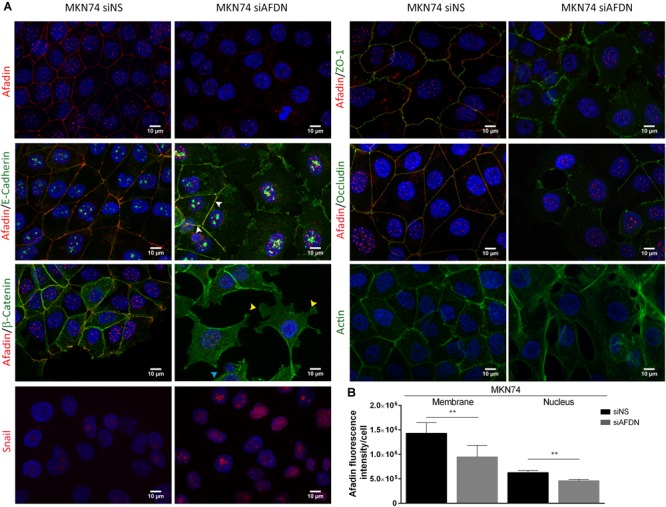
Afadin downregulation displaces apical junctional complex proteins and promotes the formation of actin stress fibers. Double immunofluorescence of Afadin (red) with AJs proteins E-cadherin and β-catenin (green), and with TJs proteins ZO-1 and occludin (green) in MKN74 cells transfected with a non-silencing siRNA (siNS) or with a siRNA to Afadin (siAFDN). Immunofluorescence of actin (green) and of Snail (red) are also shown. Nuclei were counterstained with DAPI. Scale bar, 10 μm. White arrows represent cells with Afadin not efficiently silenced by the siRNA and that retain the epithelial morphology; Yellow arrows, lamellipodia; cyan arrows, filopodia **(A)**. Quantification of Afadin fluorescence intensity in MKN74 cells in both membrane and the nucleus upon treatment with non-silencing siRNA or with a siRNA to Afadin **(B)**.

**FIGURE 4:**
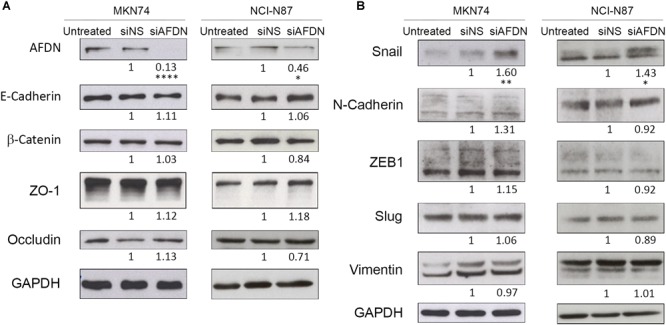
Afadin downregulation does not alter the expression of apical junctional complex proteins, but upregulates the EMT marker Snail. Western blot analyses of MKN74 and NCI-N87 cells, cells transfected with a non-silencing siRNA (siNS), or with a siRNA to Afadin (siAFDN), evaluating **(A)** apical junctional complex proteins E-cadherin, β-catenin, ZO-1, and occludin, and **(B)** mesenchymal markers Snail, N-cadherin, ZEB1, Slug, and Vimentin. GAPDH was used as loading control. Numbers below the bands correspond to quantifications. Data are representative of three independent experiments. Statistical significance was evaluated with the Student’s *t-*test.

Since Afadin is an AJC protein, we first evaluated the effect of Afadin downregulation on the localization of AJs and TJs proteins in MKN74 cells. In cells transfected with the non-silencing siRNA, the AJs proteins E-cadherin and β-catenin, and the TJs proteins occludin and ZO-1 preferentially localized at the cell membrane. In contrast, in cells where Afadin was silenced, there was an alteration of the membrane localization of E-cadherin, β-catenin, occludin, and ZO-1 (Figure 3A), without changes in the respective protein expression levels (Figure 4A). Alterations in the cellular localization of these proteins in cells where Afadin was silenced were accompanied by a striking change in the epithelial cell morphology, with loss of the actin belt, formation of actin stress fibers, and formation of lamellipodia and filopodia (Figure 3A). Interestingly, at sites where Afadin was not efficiently silenced by the siRNA, cells retained the polyhedral epithelial morphology with tight cell-to-cell contacts, contrasting with sites where Afadin expression was fully abolished by siRNA, and in which cell–cell adhesion was lost and the AJC proteins were dislocated to the cytosol (Figure 3A). Quantification of Afadin fluorescence intensity at the membrane and the nucleus in both Afadin silenced and non-silenced cells is depicted in Figure 3B. A significant decrease in Afadin expression was observed in both localizations in Afadin silenced cells, reinforcing the specificity of the Afadin siRNA and antibody.

Having shown that Afadin downregulation alters the structure of gastric cell–cell junctions and cell morphology, and knowing that Afadin negatively regulates the transcription of Snail to activate EMT in pancreatic cancer cells (Xu et al., 2015), we sought to investigate the involvement of Afadin in the EMT phenotype in the gastric context. In both MKN74 and NCI-N87 cell lines, silencing of Afadin significantly increased the levels of the EMT-associated transcription factor Snail (Figures 3A, 4B), but not of the EMT markers Slug, ZEB1, Vimentin, and N-Cadherin (Figure 4B). Overall, these results are suggestive that Afadin downregulation is implicated in the emergence of the EMT phenotype in the gastric setting.

### Afadin Downregulation Alters the TJ Barrier Function and Increases Cell Motility and Invasion

Having shown that Afadin silencing resulted in delocalization of TJ and AJ proteins from the membrane to the cytoplasm, we next addressed the functional consequences of Afadin downregulation in terms of TJ integrity, cell motility/migration, and invasion.

**FIGURE 5:**
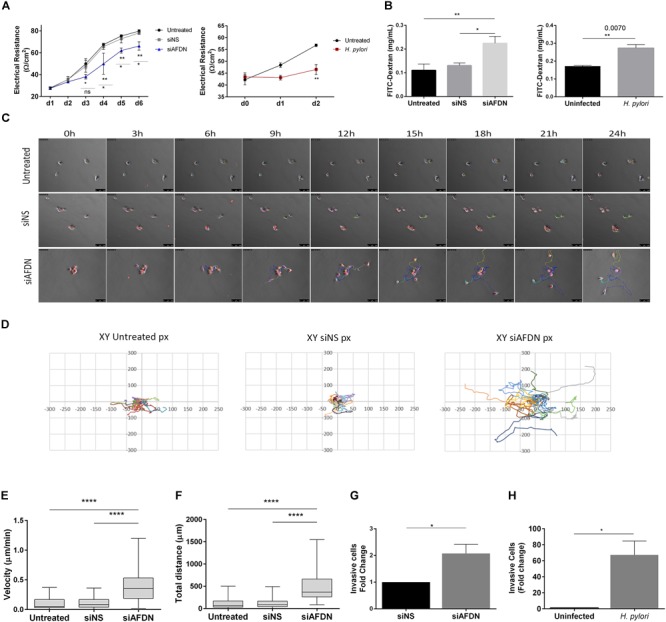
Afadin downregulation alters the TJ barrier function and increases cell motility and invasion. **(A)** Transepithelial electrical resistance (TER) measurements of MKN74 gastric cells during a period of 6 days post Afadin silencing (siAFDN) in comparison with non-silenced (siNS) and with untreated cells, and during 2 days after *H. pylori* infection in comparison with uninfected cells. **(B)** Cell monolayer permeability to 4 kDa FITC-Dextran on day 6 post Afadin silencing and on day 2 after *H. pylori* infection. **(C–F)** Single cell motility analysis: **(C)** Time-lapse microscopy photos of cells during the 24 h period. **(D)** Representative graphs of the X and Y coordinates (in pixels, px) of each cell path, on a fibronectin coated surface. **(E)** Cell velocity in μm/minute and **(F)** total distance covered by and individual cell in μm. **(G,H)** Cell invasion capacity on Matrigel-coated transwells for MKN74 cells transfected with siNS control or transfected with siAFDN **(G)**, and for MKN74 cells infected or not with *H. pylori* 26695 **(H)**. Data are presented as mean ± SEM. Statistical significance was evaluated with the one- and two-way ANOVA and with Student’s *t*-test.

To evaluate TJ integrity, cells were seeded in transwells and allowed to reach 100% confluence, after which TER was measured over six consecutive days, allowing cells to polarize. Cells where Afadin was downregulated had significantly lower electrical resistance in comparison to cells transfected with the non-silencing siRNA or to untreated cells, an effect that started to be observed from day 3 (Figure 5A). Permeability assays were performed on day 6, by measuring the permeability of the gastric monolayers to the high molecular weight 4 kDa dextran. Monolayers established with cells where Afadin expression was silenced were significantly more permeable than control cell monolayers (Figure 5B). Similar results regarding TER and permeability to FITC-4 kDa Dextran were found for MKN74 (Figures 5A,B) and NCI-N87 gastric cells (Supplementary Figures 2A,B) upon infection with *H. pylori* strain 26695.

To analyze whether there was a direct link between loss of Afadin and cell motility/migration, cells were seeded at low density, stained with the lipophilic dye CM-Dil, and tracked for 24 h with time-lapse microscopy. Analysis of the time-lapse microscopy videos revealed that, even when small groups of cells were seeded, cells with downregulation of Afadin were unable to maintain cell–cell adhesion, as opposed to untreated cells, or cells transfected with the non-silencing siRNA (Figure 5C). Coordinates of each individual cell path over the 24 h period were represented in two-dimensional graphs (Figure 5D). Silencing of Afadin significantly increased the migration velocity (Figure 5E) and the distance (Figure 5F) covered by each individual cell, compared to non-silenced or to untreated control cells. No significant differences on directionality were observed, with each cell displaying a random migration pattern.

We next addressed the involvement of Afadin in gastric cell invasion in Matrigel invasion assays. Silencing of Afadin resulted in a twofold increase in the cell invasive capacity compared to control cells transfected with the non-silencing siRNA (Figure 5G). Increased cell invasion was also observed upon infection with *H. pylori* strain 26695 in both MKN74 cells (Figure 5H) and NCI-N87 cells (Supplementary Figure 2C).

Overall, these results suggest that Afadin plays a role in gastric epithelial integrity by maintaining the structure and functions of the TJs and AJs, namely preserving the epithelial barrier and cell–cell adhesion, and suppressing cell motility and invasion.

### *H. pylori* Infection Affects the Expression and Localization of Afadin in the Human Gastric Mucosa

**FIGURE 6:**
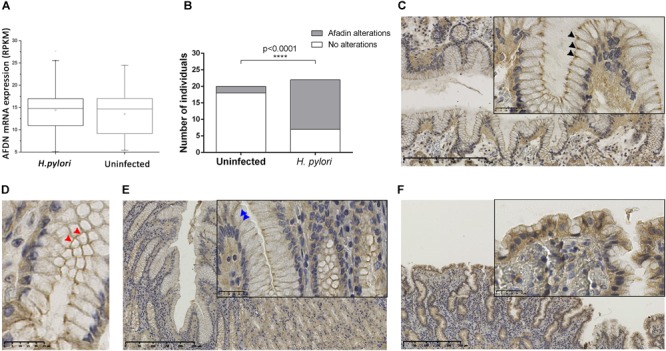
*H. pylori* infection affects the expression and localization of the Afadin protein in the human gastric mucosa. **(A)** *AFDN* gene expression in the stomach of 181 (114 uninfected and 67 *H. pylori*-infected) individuals without disease, retrieved from the GTEx database. RPKM, reads per kilobase million. Significance was evaluated with Kruskal–Wallis statistics. **(B–F)** Immunohistochemical detection of Afadin in paraffin-embedded sections of the gastric mucosa of individuals uninfected (*n* = 20) or infected with *H. pylori* (*n* = 22). **(B)** Graphical representation of Afadin alterations in the two biological groups. Statistical significance was determined with the Fisher’s exact test. Representative micrographs of Afadin staining in an uninfected individual, showing a strong intensity at the apicolateral epithelial cell–cell contacts (black arrowheads) **(C)**, and a belt-like pattern (red arrowheads) **(D)** in longitudinal and cross sections, respectively, and in *H. pylori*-infected individuals, presenting decreased intensity/loss of membrane staining at the cell–cell contacts (blue arrowheads) **(E)** or showing cytoplasmic staining in areas of epithelial tufting **(F)**.

To determine the impact of *H. pylori* infection on Afadin *in vivo*, we first evaluated *AFDN* gene expression using the data available at the GTEx database. Data was retrieved from gastric samples of 181 individuals without disease, and 67 individuals (37%) were identified to be infected with *H. pylori*. No statistically significant differences (*p* = 0.30) were observed between *AFDN* gene expression levels in uninfected and *H. pylori*-infected patients (Figure 6A).

The expression of Afadin was also evaluated by immunohistochemistry in a series of 42 tissue specimens that have been characterized regarding the histopathological features of the gastric mucosa and the presence of *H. pylori* infection (Supplementary Table 1). This series comprised 20 cases with a normal gastric mucosa, which were negative for *H. pylori*, and 22 cases with chronic gastritis, all infected with *H. pylori.* Only one case had glandular atrophy and intestinal metaplasia. As shown in Figure 6B, the majority of the cases that had a non-infected gastric mucosa exhibited Afadin staining with a belt-like pattern with strong intensity dots at the apicolateral epithelial cell–cell contacts (18/20; 90%; Figures 6C,D). Alterations to the normal pattern of Afadin staining were significantly more frequent in *H. pylori*-infected (15/22; 68%) than in uninfected individuals (2/20; 10%; *p* < 0.0001). These modifications comprised decreased intensity/loss of membrane staining at cell–cell contacts (observed in 12 of the *H. pylori*-infected and in two uninfected individuals, Figure 6E), and loss of staining at cell–cell contacts and cytoplasmic staining in areas of epithelial tufting (observed in three *H. pylori*-infected individuals, Figure 6F). No statistical significant relationships were observed between polymorphonuclear activity and Afadin alterations.

The results obtained in gastric tissues validate our findings in cell lines and support that *H. pylori* infection alters Afadin protein expression and localization *in vivo.*

## Discussion

In the present study, we evaluated the effect of *H. pylori* infection on the apical-junctional complex protein Afadin. Our results in two different *in vitro* infection models showed that *H. pylori* reduces Afadin expression *in vitro*, and this effect was independent of the major virulence factors CagA and VacA, of factors delivered *via* the bacterial T4SS, and of soluble bacterial factors secreted to the extracellular medium. In the gastric cell lines that we have used, we have identified Afadin at the cell membrane in a continuous pattern, and in the nucleus in a speckled pattern. The reduction in Afadin expression upon *H. pylori* infection was observed for both locations. Afadin has two major splicing isoforms, the l- and the s-Afadin, the former has an additional F-actin binding domain and is unable to localize to the nucleus, whilst the latter lacks that domain and is a dual residency protein (Buchert et al., 2007). Interestingly, other cytoplasmic protein components of cell–cell junctions are able to shuttle to the nucleus to convey signal events from the cell membrane (Harris and Lim, 2001). Still, the functions and dynamics of the nuclear s-Afadin are largely unknown, as is the role of *H. pylori* infection in this context.

In the human gastric mucosa, we also observed an association between *H. pylori* infection and modifications to the normal pattern of Afadin, encompassing decreased intensity or loss of membrane staining at the apicolateral epithelial cell–cell contacts and, less frequently, cytoplasmic staining in areas of epithelial tufting. The observation that *H. pylori*-infected and uninfected individuals do not have differences in gastric *AFDN* gene expression, as it was seen in MKN74 gastric cells, suggests that Afadin changes by *H. pylori* occur post-transcriptionally.

Activation of an EMT program has been proposed as the critical mechanism for the acquisition of malignant phenotypes by epithelial cells (Moreno-Bueno et al., 2008). The key characteristics of EMT include disruption of junctional contacts, cell cytoskeleton reorganization, and acquisition of motility/migration, which are accompanied by the expression of mesenchymal markers and repression of epithelial markers (Lamouille et al., 2014). In keeping with these features, in cells that were infected with *H. pylori* there was a downregulation of epithelial properties, by displacement from cell–cell contacts and decrease in protein expression of multiple components of the TJs and of the AJs, including Afadin. Parallel to the remodeling of the junctional complexes, in *H. pylori*-infected cells, there were significant increases in the protein expression of EMT markers ZEB1, Vimentin, Slug, N-cadherin, and Snail. Importantly, these effects were observed in cell lines that establish competent junctional complexes. Functionally, *H. pylori* infection impaired TJs function and increased cell invasion capacity. Our results are in agreement with earlier data linking *H. pylori* with the induction of EMT-like morphological changes and of mesenchymal gene expression (Saito et al., 2010; Baud et al., 2013; Lee et al., 2014; Yu et al., 2014; Wroblewski et al., 2015). In contrast, our data show that *H. pylori-*mediated EMT through Afadin downregulation is independent of CagA, T4SS, and VacA. This suggests that *H. pylori* triggers EMT at different extents by several pathways and bacterial factors. In fact, ectopic expression of CagA in MDCK cells increased only fibronectin and vimentin mesenchymal markers, without downregulation of epithelial markers (Saito et al., 2010). In another report, CagA induces EMT with increased expression of β-catenin and Snail with loss of E-cadherin (Lee et al., 2014). Also, CagA was shown to promote activation of TWIST1 and vimentin, and inhibition of E-cadherin, without changes in Snail expression (Yu et al., 2014).

We observed that silencing of Afadin expression by RNAi led to significant displacement of proteins from the junctional complexes to the cytoplasm, accompanied by morphological changes, with loss of the actin belt, formation of actin stress fibers, and formation of lamellipodia and filopodia. Additionally, Afadin downregulation increased gastric cell motility and invasion, and notably increased the expression of Snail. In a model of pancreatic cancer, Afadin regulates Snail expression by antagonizing the interaction between Dvl2 and FOXE1. In case of low levels of Afadin, Dvl2 becomes available to bind and enhance FOXE1, activating Snail expression (Xu et al., 2015). Since our results also show downregulation of Afadin with increased Snail expression, this could be an alternative and yet undescribed mechanism through which *H. pylori* promotes EMT in epithelial cells. Interestingly, in this model the nuclear localization of Afadin was required for the repression of Snail (Xu et al., 2015). This may suggest that the decrease of nuclear Afadin that we observe upon *H. pylori* infection underlies the increase in Snail expression. Further studies are needed to explore the signaling pathways leading to EMT mediated by Afadin in the context of *H. pylori* infection. Our findings are also in agreement with data showing a role for Afadin in the regulation of breast cancer cell migration and invasion (Fournier et al., 2011).

## Conclusion

In conclusion, our findings suggest that Afadin contributes to maintaining gastric epithelial junctional structures and to suppressing EMT, features that are lost during *H. pylori* infection and may contribute to gastric carcinogenesis.

## Author Contributions

MM and ML conceptualized and designed the study. MM, JM, BC, NM, LP, FC, CF, and ML acquired the data. MM, BC, FC, LP, CF, and ML performed the data analysis and interpreted the data. All authors drafted the manuscript or revised it critically for important intellectual content.

## Conflict of Interest Statement

The authors declare that the research was conducted in the absence of any commercial or financial relationships that could be construed as a potential conflict of interest.

## Footnotes

1 http://imagej.net/
