# Supplementary material for: Afadin Downregulation by Helicobacter pylori Induces Epithelial to Mesenchymal Transition in Gastric Cells
Source: Front Microbiol. 2018 Nov 9;9:2712. doi: 10.3389/fmicb.2018.02712 (PMC6237830; doi:10.3389/fmicb.2018.02712)
Supplement: Supplementary file 1 [file Data_Sheet_1.pdf]

frontiers

Supplementary Material

Afadin downregulation by *Helicobacter pylori* induces epithelial to mesenchymal transition in gastric cells

Miguel Sardinha Marques[1,2,3], Joana Melo[1,2,4], Bruno Cavadas[1,2,4], Nuno Mendes1[,2], Luísa Pereira[1,2,3], Fátima Carneiro[1,2,3,5], Ceu Figueiredo[1,2,3,6,] *Marina Leite[1,2,3,6]

[1] i3S – Instituto de Investigação e Inovação em Saúde, Universidade do Porto, Porto, Portugal

[2] Ipatimup – Institute of Molecular Pathology and Immunology of the University of Porto, Porto, Portugal

[3] Faculty of Medicine of the University of Porto, Department of Pathology, Porto, Portugal

[4] Instituto de Ciências Biomédicas Abel Salazar (ICBAS), University of Porto, Porto, Portugal

[5] Centro Hospitalar São João, Department of Pathology, Porto, Portugal

[6] Shared co-authorship.

*** Correspondence:** Ceu Figueiredo: cfigueiredo@ipatimup.pt

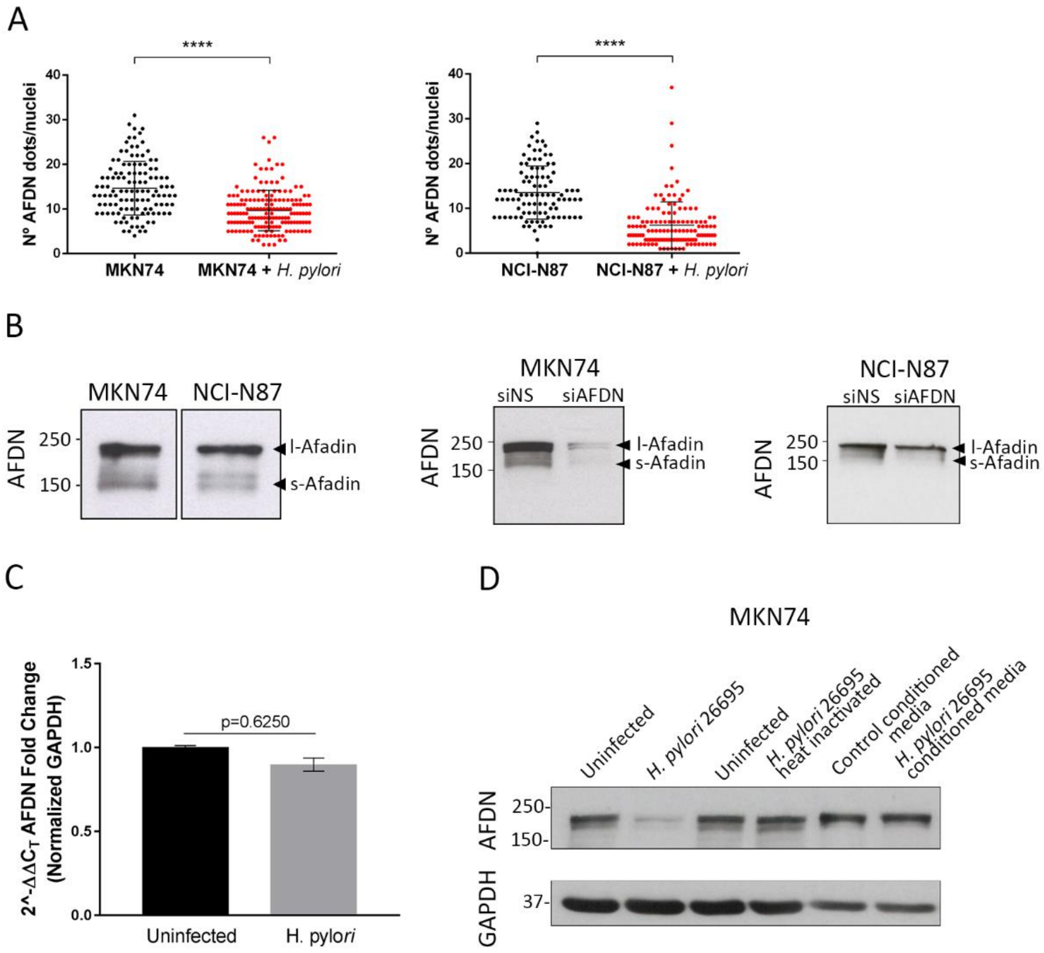

Supplementary Figure 1. (**A)** Quantification of the Afadin nuclear dots from immunofluorescence of MKN74 and NCI-N87 gastric cells, untreated or infected with *H. pylori* strain 26695. Data correspond to the mean value ± SEM and are representative of at least 3 independent experiments. Statistical significance was evaluated with the Student's t-test. **(B)** Detection of both long (~220 kDa)- and short (~150 kDa)-Afadin isoforms by western blot analysis, using the anti-AF6 antibody (Clone 35/AF6, #610732, BD Transduction Laboratories™), in untreated, non-silencing siRNA (siNS)-, or siRNA to Afadin (siAFDN)-treated MKN74 and NCI-N87 gastric cell lines. **(C)** Afadin mRNA expression in MKN74 gastric cells uninfected and infected with *H. pylori* strain 26695 for 24 hours, by quantitative real-time PCR using the $\Delta\Delta C_T$ method. Data represent relative expression changes (fold change) of the mean ± standard deviation of four independent experiments relative to uninfected control and normalized to GAPDH endogenous control. Statistical significance was evaluated with the Wilcoxon test ($p<0.05$). **(D)** Western blot of MKN74 cells infected with viable and heat-inactivated (95 ºC, 10 min) *H. pylori* strain 26695, and with bacterial conditioned media from a 24 hours *H. pylori* liquid culture.

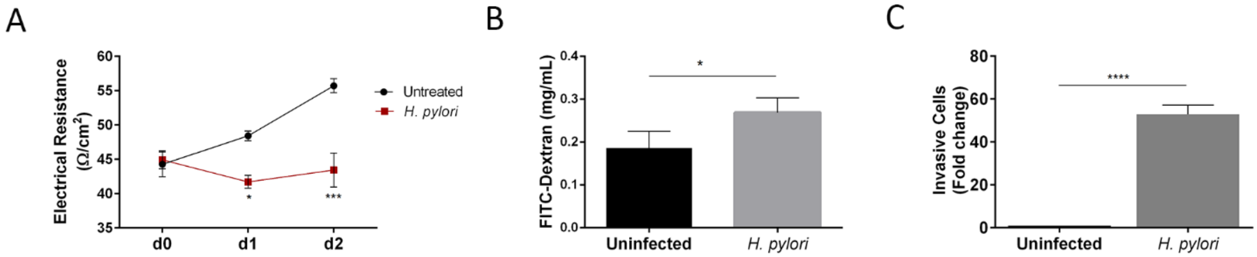

Supplementary Figure 2. Evaluation of barrier function and invasive capacity of NCI-N87 gastric cells upon *H. pylori* infection. **(A)** Transepithelial electrical resistance (TER) measurements of gastric cells NCI-N87 for 2 days upon *H. pylori* infection. **(B)** Cell monolayer permeability to 4kDa FITC-Dextran on day 2 upon *H. pylori* infection. **(C)** Cell invasion capacity on Matrigel-coated transwells for NCI-N87 cells uninfected or infected with *H. pylori* strain 26695. Data are presented as mean ± SEM. Statistical significance was evaluated with the two-way ANOVA and with Student's t-test.

Supplementary Table 1. Clinico-pathological patient data.

Case No.	Sex	Age	Chronic gastritis	Polymorphonuclear activity	Atrophy/intestinal metaplasia	*H. pylori*
1	F	34	Present	Absent	Absent	Positive
2	F	58	Present	Absent	Absent	Positive
3	F	40	Present	Present	Present	Positive
4	F	44	Present	Absent	Absent	Positive
5	F	46	Present	Absent	Absent	Positive
6	F	39	Present	Absent	Absent	Positive
7	F	37	Present	Absent	Absent	Positive
8	F	36	Present	Absent	Absent	Positive
9	F	39	Present	Present	Absent	Positive
10	F	44	Absent	Absent	Absent	Negative
11	F	32	Absent	Absent	Absent	Negative
12	F	63	Present	Present	Absent	Positive
13	F	61	Present	Present	Absent	Positive
14	M	42	Absent	Absent	Absent	Negative
15	F	31	Absent	Absent	Absent	Negative
16	F	37	Present	Present	Absent	Positive
17	F	59	Absent	Absent	Absent	Negative
18	F	29	Absent	Absent	Absent	Negative
19	F	30	Absent	Absent	Absent	Negative
20	F	37	Present	Present	Absent	Positive
21	F	15	Absent	Absent	Absent	Negative
22	F	57	Present	Present	Absent	Positive
23	F	24	Absent	Absent	Absent	Negative
24	F	41	Absent	Absent	Absent	Negative
25	F	60	Absent	Absent	Absent	Negative
26	M	35	Absent	Absent	Absent	Negative
27	M	36	Present	Absent	Absent	Positive
28	F	31	Present	Absent	Absent	Positive
29	F	50	Absent	Absent	Absent	Negative
30	F	53	Present	Absent	Absent	Positive
31	F	37	Absent	Absent	Absent	Negative
32	F	51	Absent	Absent	Absent	Negative
33	F	41	Present	Present	Absent	Positive
34	M	20	Present	Present	Absent	Positive
35	F	17	Absent	Absent	Absent	Negative
36	F	51	Absent	Absent	Absent	Negative
37	M	51	Present	Absent	Absent	Positive
38	F	44	Present	Absent	Absent	Positive
39	F	61	Absent	Absent	Absent	Negative
40	M	20	Absent	Absent	Absent	Negative
41	F	39	Present	Present	Absent	Positive
42	F	24	Absent	Absent	Absent	Negative
